# Supplementary material for: Identification and functional analysis of non-coding regulatory small RNA FenSr3 in Bacillus amyloliquefaciens LPB-18
Source: PeerJ. 2023 May 15;11:e15236. doi: 10.7717/peerj.15236 (PMC10194069; doi:10.7717/peerj.15236)
Supplement: Supplemental Information 4 [file peerj-11-15236-s004.zip › KO/CK-vs-T1_map/map05162.html]

KEGG PATHWAY: Measles - Reference pathway


|  |  |
| --- | --- |
| **Measles - Reference pathway** |  |

[
Pathway menu
| Organism menu
| Pathway entry
| Show description
| User data mapping
]

Measles virus (MV) is highly contagious virus that leads infant death worldwide. Humans are the unique natural reservoir for this virus. It causes severe immunosuppression favouring secondary bacterial infections. Several MV proteins have been suggested to disturb host immunity. After infection of host lymphoid cells via SLAM, MV inhibits cytokine response by direct interference with host signaling systems. Three proteins (P, V, and C) associate with Jak/STAT proteins in interferon-triggered pathway and other important proteins related to apoptosis. Interaction between MV and host brings about the shift towards a Th2 response by decreasing IL-12 production and induces lymphopenia by suppressing cell proliferation.

|  |  |  |
| --- | --- | --- |
| Reference pathway | 184% 150% 122% 100% 82% 67% 55% | 图片下载 |
